# Radial incision and balloon dilation with triamcinolone filling for the severe rectal anastomotic stricture

**DOI:** 10.1055/a-2742-5758

**Published:** 2025-11-28

**Authors:** Yusuke Takahashi, Kotaro Shibagaki, Yui Fukada, Satoshi Kotani, Tomotaka Yazaki, Norihisa Ishimura, Shunji Ishihara

**Affiliations:** 1 175764 Department of Gastroenterology, Faculty of Medicine, Shimane University, Izumo, Japan

A man in his 60s underwent open low anterior resection with diverting ileostomy for advanced rectal cancer. Six months later, colonoscopy revealed a pinhole-like anastomotic stricture ( Fig. 1 **a** ), and contrast enema demonstrated a 5-cm tortuous stenosis extending proximally, with narrowings at both the anal and oral sides ( Fig. 1 **b** ). Because conventional balloon dilation was considered to carry a high risk of perforation and recurrence, we treated the stricture using a combined radial incision and balloon dilation technique ( Video 1 ), with a schematic overview of the procedure provided in Fig. 2 .

**Fig. 1:**
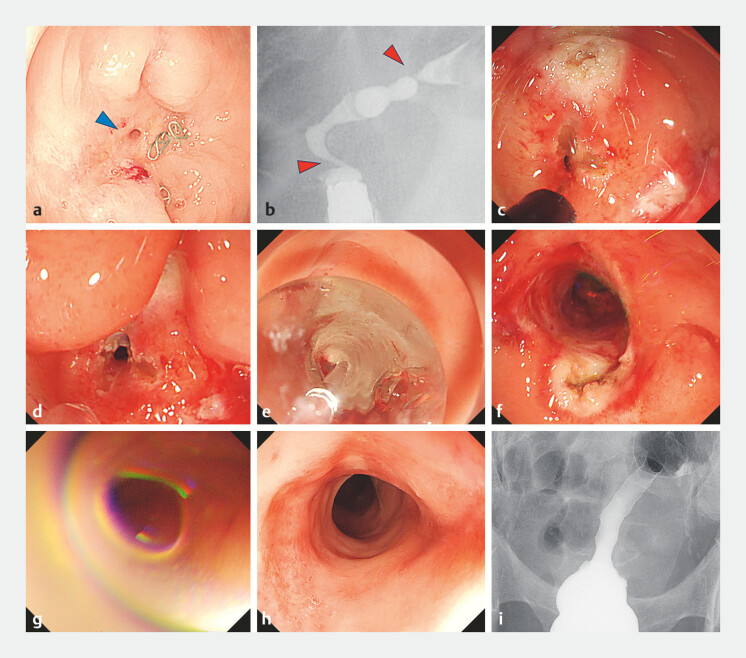
Sequential endoscopic and radiographic findings during rectal anastomotic stricture treatment. **a** A pinhole-like anastomotic stricture after rectal cancer surgery (blue arrow) with exposed staples. **b** Contrast enema revealed a 5-cm tortuous anastomotic stricture with two severe narrowings at the anal and oral ends. **c** After staple removal using a HookKnife, radial incisions were made in the anal-side stricture. **d** Four radial incisions were created. **e** Balloon dilation was performed to a diameter of 15 mm at 8 atm. **f** Multiple mucosal lacerations allowed scope passage through the anal-side stricture, and the same procedure was applied to the oral side. **g** The lumen was filled with 80 mg of triamcinolone acetonide solution to prevent restenosis. **h** An endoscopic view at 7 weeks showing complete epithelialization with adequate scope passage. **i** Contrast enema at 7 weeks demonstrating marked improvement of the stricture compared with baseline.

**Fig. 2:**
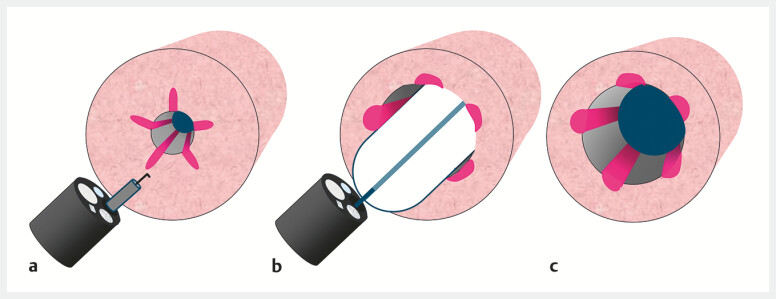
Schematic of radial incision and balloon dilation for the severe anastomotic stricture. **a** Radial incisions were created using a high-frequency knife. **b** Balloon dilation was performed to a diameter of 15 mm at 8 atm. **c** Multiple lacerations created by the incisions allowed effective stricture expansion while minimizing the risk of perforation.

Radial incision and balloon dilation with triamcinolone filling for the severe rectal anastomotic stricture.Video 1

First, exposed staples were removed with a HookKnife (Olympus, Tokyo, Japan). Second, multiple radial mucosal incisions were created ( Fig. 1 **c, d** ). Third, balloon dilation was performed with a wire-guided through-the-scope balloon (CRE PRO GI; Boston Scientific, Marlborough, MA, USA) to a diameter of 15 mm at 8 atm ( Fig. 1 **e** ), which allowed smooth passage of the endoscope ( Fig. 1 **f** ). The same procedure was subsequently applied to the oral-side stricture, resulting in successful resolution of both strictures ( Fig. 1 **f** ). Finally, 80 mg of triamcinolone acetonide (TA) solution was instilled into the lumen to prevent restenosis ( Fig. 1 **g** ). The procedure was repeated once at 3 weeks for the anal side stricture and at 5 weeks for the oral side stricture, respectively. By 7 weeks, complete epithelialization without the recurrent stricture was confirmed both by endoscopy ( Fig. 1 **h** ) and contrast enema ( Fig. 1 **i** ), after which stoma closure was performed.

Radial incision allows balloon dilation to create multiple lacerations, providing effective expansion while avoiding excessive focal stress on a single site and thereby reducing the risk of perforation. Although TA injection after balloon dilation is generally considered effective 1 , in this case, the stricture was long and firm, making local injection difficult; therefore, we applied the TA filling method previously reported by our group in the esophagus 2 3 4 , which resulted in a favorable outcome.

Endoscopy_UCTN_Code_TTT_1AQ_2AF

## Notes

Endoscopy E-Videos https://eref.thieme.de/e-videos
*E-Videos* is an open access online section of the journal *Endoscopy* , reporting on interesting cases and new techniques in gastroenterological endoscopy. All papers include a high-quality video and are published with a Creative Commons CC-BY license. Endoscopy E-Videos qualify for HINARI discounts and waivers and eligibility is automatically checked during the submission process. We grant 100% waivers to articles whose corresponding authors are based in Group A countries and 50% waivers to those who are based in Group B countries as classified by Research4Life (see: https://www.research4life.org/access/eligibility/ ). This section has its own submission website at https://mc.manuscriptcentral.com/e-videos .
